# Loss of SynDIG1 Reduces Excitatory Synapse Maturation But Not Formation *In Vivo*

**DOI:** 10.1523/ENEURO.0130-16.2016

**Published:** 2016-10-21

**Authors:** George Chenaux, Lucas Matt, Travis C. Hill, Inderpreet Kaur, Xiao-Bo Liu, Lyndsey M. Kirk, David J. Speca, Samuel A. McMahon, Karen Zito, Johannes W. Hell, Elva Díaz

**Affiliations:** 1 Department of Pharmacology, University of California, Davis, California 95616; 2 Center for Neuroscience, University of California, Davis, California 95616

**Keywords:** AMPA receptor, excitatory synapse, hippocampus, synapse development, synapse maturation, *SynDIG1*

## Abstract

Modification of the strength of excitatory synaptic connections is a fundamental mechanism by which neural circuits are refined during development and learning. Synapse Differentiation Induced Gene 1 (SynDIG1) has been shown to play a key role in regulating synaptic strength *in vitro*. Here, we investigated the role of SynDIG1 *in vivo* in mice with a disruption of the *SynDIG1* gene rather than use an alternate loxP-flanked conditional mutant that we find retains a partial protein product. The gene-trap insertion with a reporter cassette mutant mice shows that the *SynDIG1* promoter is active during embryogenesis in the retina with some activity in the brain, and postnatally in the mouse hippocampus, cortex, hindbrain, and spinal cord. Ultrastructural analysis of the hippocampal CA1 region shows a decrease in the average PSD length of synapses and a decrease in the number of synapses with a mature phenotype. Intriguingly, the total synapse number appears to be increased in *SynDIG1* mutant mice. Electrophysiological analyses show a decrease in AMPA and NMDA receptor function in *SynDIG1*-deficient hippocampal neurons. Glutamate stimulation of individual dendritic spines in hippocampal slices from SynDIG1-deficient mice reveals increased short-term structural plasticity. Notably, the overall levels of PSD-95 or glutamate receptors enriched in postsynaptic biochemical fractions remain unaltered; however, activity-dependent synapse development is strongly compromised upon the loss of SynDIG1, supporting its importance for excitatory synapse maturation. Together, these data are consistent with a model in which SynDIG1 regulates the maturation of excitatory synapse structure and function in the mouse hippocampus *in vivo*.

## Significance Statement

Synapse Differentiation Induced Gene 1 (SynDIG1) is a brain-specific transmembrane protein that is enriched at excitatory synapses. Removal of endogenous SynDIG1 in the mouse brain results in a significant reduction in excitatory synapse maturation, as determined by a combination of structural and electrophysiological approaches. In contrast, biochemical analysis reveals that excitatory synapse composition is unchanged upon the loss of SynDIG1. Together, these data support a model in which SynDIG1 is important for excitatory synapse maturation but is not required for synaptogenesis *in vivo*.

## Introduction

The mammalian CNS enables sophisticated computation and behavior due to the development of a staggeringly complex neuronal network (Jessell and Kandel, 1993). This circuitry depends upon precise cell–cell communication, which occurs at the synapse. Glutamate released by the presynaptic cell activates NMDA, AMPA, and/or kainate-type ionotropic glutamate receptors. The majority of synapses are formed postnatally (Waites et al., 2005; McAllister, 2007), yet synapses remain plastic and can be formed, lost, strengthened, or weakened throughout adulthood. Functional excitatory synapse maturation is in part dependent on the directed trafficking of AMPA receptors to postsynaptic sites, which has been the topic of intense investigation (Malinow and Malenka, 2002; Bredt and Nicoll, 2003; Chen et al., 2007). Synaptic targeting of AMPA receptors is regulated by intracellular scaffolding proteins (Elias and Nicoll, 2007) as well as by a diverse array of transmembrane accessory proteins (Jackson and Nicoll, 2011; Haering et al., 2014).

Synapse Differentiation Induced Gene I (SynDIG1) was identified in a screen comparing gene expression profiles from the cerebella of wild-type (WT) animals to *Lurcher* mice, which display defects in Purkinje neuronal differentiation (Díaz et al., 2002). SynDIG1 is a highly conserved type II integral membrane protein with a large intracellular N-terminal region followed by a single transmembrane domain and a second hydrophobic segment that does not span the membrane (Kalashnikova et al., 2010). SynDIG1 overexpression in cultures of dissociated rat hippocampal neurons results in a significant increase in excitatory synapse strength and number, as reflected by increased postsynaptic density (PSD)-95 and AMPA receptors at excitatory synapses while the knockdown of SynDIG1 results in a decrease of synaptic PSD-95 and AMPA receptor levels (Kalashnikova et al., 2010). There is conflicting evidence concerning the regulation of NMDA receptors by SynDIG1. In cultures of dissociated rat hippocampal neurons, NMDA receptor-dependent miniature EPSC (mEPSC) and synaptic NMDA receptor content were unchanged upon SynDIG1 overexpression or knockdown (Kalashnikova et al., 2010), suggesting that SynDIG1 selectively regulates synaptic strength by altering the levels of AMPA receptors and PSD-95 at synapses. However, shRNA targeted to SynDIG1 in hippocampal slice culture resulted in decreased mEPSC frequency, a trend toward decreased mEPSC amplitude and decreased AMPA receptor and NMDA receptor synaptic transmission (Lovero et al., 2013), suggesting a more general role in excitatory synapse development. One explanation for these differences might reflect the efficiency of the knockdown of SynDIG1 in dissociated hippocampal neurons compared with hippocampal slice culture. For example, in hippocampal slice culture, SAP-102 knockdown via biolistic delivery of shRNA reduced both AMPAR and NMDAR currents, while SAP-102 knockdown via viral delivery of shRNA had no effect on AMPAR current and provided a small decrease in NMDAR current (Levy et al., 2015), suggesting that biolistic delivery might be more efficient at knockdown compared with viral delivery.

Given the differences in the ability of SynDIG1 to regulate excitatory synapses and modulate glutamate receptor function in dissociated hippocampal neurons and in slice culture, it is essential to understand when and how SynDIG1 functions during neural development within the intact brain. To rigorously address these issues, we established and characterized SynDIG1 mutant mice. Here we demonstrate that mice with reduced SynDIG1 exhibit deficits in the ultrastructure and function of mature excitatory synapses. The overall reduction in the number of mature synapses by multiple criteria suggests that SynDIG1 is important for development. In contrast, biochemical fractionation studies indicate no significant differences in synapse composition. Together, SynDIG1 appears to play an important role in the ability of excitatory synapses to reach or sustain maturity *in vivo* without gross changes to normal synapse composition.

## Materials and Methods

### Characterization of a conditional SynDIG1^flox^ allele

All animal procedures followed National Institutes of Health (NIH) guidelines and have been approved by the Institutional Animal Care and Use Committee at the University of California, Davis. Preliminary analyses were performed on a conditional *SynDIG1* allele generated by the Mouse Biology Program at the University of California, Davis, referred to here as *SynDIG1^flox^*. A targeting vector harboring a neomycin resistance (neo) cassette flanked by FRT sites and loxP sites flanking exon 4 was electroporated into mouse embryonic stem cells. Flp recombinase exposure *in vitro* excised the neo cassette. Molecular analyses were performed on a mixed C57BL/6N and C57BL/6J genetic background. For RNA analysis, *SynDIG1^flox/+^* heterozygous mice were crossed with a nestin Cre driver line [Tg(Nes-cre)1Kln; RRID:IMSR_JAX:003771] generating heterozygous mice with Nes-cre^+^ cells in which exon 4 was deleted (*SynDIG1^Δexon4/+^*). Subsequent breeding generated homozygous mutant mice (*SynDIG1^Δexon4/Δexon4^*), heterozygous mutant mice (*SynDIG1^Δexon4/+^*), and homozygous wild-type mice (*SynDIG1^+/+^*) that expressed the Cre transgene. For immunoblot analysis, *SynDIG1^flox/+^* heterozygous mice were crossed with a CMV-cre driver line [B6.C-Tg(CMV-cre)1Cgn/J; RRID:IMSR_JAX:006054] generating heterozygous mice with CMV-cre^+^ cells in which exon 4 was deleted (*SynDIG1^Δexon4/+^*). Subsequent breeding of heterozygous *SynDIG1^Δexon4/+^* mice generated homozygous mutant mice (*SynDIG1^Δexon4/Δexon4^*), heterozygous mutant mice (*SynDIG1^Δexon4/+^*), and homozygous wild-type mice (*SynDIG1^+/+^*) that expressed the Cre transgene. In all experiments, *SynDIG1* mutant mice were compared with mice with the unmodified wild-type *SynDIG1* allele.

### Generation of *SynDIG1* ^β-gal^ mutant mice and subsequent breeding

The gene-trap allele [*SynDIG1* ^Gt(IST12733A12)Tigm^] was generated by the Texas A&M Institute for Genomic Medicine (TIGM; RRID:IMSR_TIGM:IST12733A12). The Victr 76 viral plasmid was used to insert a splice acceptor, a β-geo (β-galactosidase/neomycin) cassette, and a synthetic polyA signal/transcriptional blocker all flanked by two viral long-terminal repeat segments in between exons 4 and 5 of the *SynDIG1* locus. Mice were housed under IACUC standards and were maintained in the C57BL/6 strain. Embryonic mouse ages are defined as embryonic day 0.5 (E0.5) upon first discovery of a vaginal plug. The day of birth is referred to as postnatal day 0 (P0). These mice are referred to as *SynDIG1* ^β-gal^ mutants.

Splicing to the inserted sequence in the SynDIG1 trapped allele is predicted to generate a transcript that encodes 5 aa (VEEQA) followed by a stop codon immediately after the SynDIG1 transmembrane domain encoded by exon 4 at position 206, thereby resulting in a SynDIG1 mutant protein composed of 211 aa with a calculated molecular weight of 23.6 kDa. Immunoblot analysis with anti-SynDIG1 antibody (epitope within amino acids 19–43) does not detect a protein in this size range, suggesting that the mutant SynDIG1 protein is not stably expressed. A fusion protein between SynDIG1 and β-geo is not predicted based on sequence information. Consistently, immunoblot analysis with antibodies against β-galactosidase (β-gal) recognizes bands at ∼150 kDa that are not recognized by the SynDIG1 antibodies. Therefore, β-gal expression is likely driven by the SynDIG1 promoter, and this reporter protein is mostly retained within the cell body in *SynDIG1* ^β-gal^ mutants.

### Total RNA isolation and RT-PCR

Total RNA was isolated from adult brain tissue using TRIzol reagent (Invitrogen). Superscript III (ThermoFisher Scientific) was used for first strand synthesis. Subsequent PCR amplification utilized two primers (forward: CAC CAA AGA CAG CCT GGA GT) and (reverse: GGC CTG ATG GAA GTC TCC TT), which annealed to the sequence in exon 3 and exon 5, respectively, to amplify products of 295 or 138 bp, respectively, in wild-type or *SynDIG1^Δexon4^*transcripts.

### Biochemical fractionation and immunoblotting

For characterization of protein expression in *SynDIG1^flox^* and *SynDIG1* ^β-gal^ animals, brain tissue was homogenized in a dounce homogenizer in 0.32 m sucrose and 1 mm HEPES, pH 7.4, with protease inhibitors (leupeptin, aprotinin, pepstatin A, and phenylmethylsulfonyl fluoride). Lysate was spun for 10 min at 10,000 × *g*, and the resultant crude synaptosomal membrane fraction was used for immunoblot analysis, as described below.

For biochemical fractionation, the rostral two-thirds of mouse brains were homogenized in a dounce homogenizer in 0.32 m sucrose, 1 mm Tris, pH 7.4, and 1 mm MgCl_2_ with protease inhibitors (leupeptin, aprotinin, pepstatin A, and phenylmethylsulfonyl fluoride). Lysate was spun for 15 min at 1400 × *g*. Supernatant was collected and saved. The pellet was homogenized with more sucrose solution, and large insoluble debris and the nuclear fraction were removed by centrifugation at 710 × *g*. The supernatants were pooled, and an aliquot was saved as the postnuclear supernatant (S1) fraction. The remaining S1 was centrifuged at 16,000 × *g*, and the pellet was collected as the membrane-enriched fraction (P2), while the supernatant was considered the cytoplasmic protein-enriched supernatant (S2) fraction. The P2 fraction was resuspended in a 0.32 m sucrose solution without MgCl_2_, and was layered over 0.85, 1.0, and 1.25 m sucrose gradients, spun at 40,000 × *g* for 2 h, and synaptosomal-enriched fraction (Syn) was collected between 1.0/1.25 m gradients. Triton X-100 was added to Syn to a final concentration of 0.5% and incubated for 15 min at 4°C. The pellet was collected after spinning at 100,000 × *g* for 30 min. An aliquot of the supernatant was saved as a presynaptic fraction containing synaptic vesicle proteins and other presynaptic proteins [synaptic vesicle- and presynaptic-enriched (S3) fraction]. The pellet was resuspended in sucrose buffer, placed over another sucrose gradient (1.0/1.5/2.0 m), centrifuged at 40,000 × *g* for 2 h, and the second gradient layer was collected in 1.5/2.0 m sucrose. This fraction was exposed to Triton X-100 again, and a final PSD pellet was collected after centrifugation at 100,000 × *g* for 1 h, with the pellet resuspended via sonication to obtain the PSD-enriched fraction (PSD).

Protein levels were quantified using a BCA assay (ThermoScientific Pierce). The 10–20 μg of each biochemical fraction was separated on SDS-PAGE gels ranging from 7% to 15%. Proteins were transferred onto nitrocellulose membranes and blocked with 5% nonfat milk powder in 1× PBS. Membranes were blotted with the following primary antibodies: mouse antibodies against PSD-95 [catalog #75-028, NeuroMab (RRID:AB_2292909)], synaptophysin [catalog #101011, Synaptic Systems (RRID:AB_887824)], SynDIG1 [catalog #75-251, NeuroMab (RRID:AB_10999753)], GluA2 [catalog #75-002, NeuroMab (RRID:AB_2232661)], PSD-93 [catalog #75-057, NeuroMab (RRID:AB_2277296)], β-tubulin [catalog #05-661, Millipore (RRID:AB_309885)], GluN1 [catalog #556308, BD Biosciences (RRID:AB_396353)], and Pick1 [catalog #73-040, NeuroMab (RRID:AB_10672986)]; rabbit antibodies against SynDIG4 (Anti-Prrt1, catalog #17261-1-AP, ProteinTech), β-actin [catalog #ab8224, Abcam (RRID:AB_449644)], GluA1 [catalog #AB1504, Millipore (RRID:AB_2113602)], and GluN2B (provided by J.W.H.; Lim et al., 2002); and guinea pig antibodies against v-Glut1 [catalog #AB5905, Millipore (RRID:AB_2301751)]. For chemiluminescence, goat anti-mouse horseradish peroxidase was used with SuperSignal West Pico substrate (ThermoScientific Pierce). For quantitative analyses, goat anti-rabbit (IR680) and goat anti-mouse (IR800) secondary antibodies (LI-COR) were used. Protein concentrations were quantified on an Odyssey LI-COR system using ImageStudio software. S1, P2, Syn, and PSD fractions were normalized to β-actin or β-tubulin. Then, the normalized protein level of *SynDIG1* ^β-gal^ mutant fraction was divided by normalized WT protein levels to determine the relative change in protein. Fractionations were performed in triplicate. Quantification data were grouped by fractionation prior to statistical analysis. No significant difference was found using *t* test analyses.

### X-gal staining

Unfixed fresh mouse brains and vertebrae were quickly frozen on dry ice in Optimal Cutting Temperature (OCT) medium, and cut in 15–25 µm sections on a Leica cryostat. Sections were mounted on Superfrost Plus slides, dried, washed in 0.1 m phosphate buffer, pH 7.3, with 2 mm MgCl_2_ and 0.02% Nonidet P-40, and then stained overnight in 1.0 mg/ml X-gal (5-bromo-4-chloro-3-indolyl-β-d-galactopyranoside). Sections were washed, fixed in 4% paraformaldehyde, washed, counterstained with nuclear fast red, dehydrated with ethanol, and mounted. These sections were imaged on an AxioPlan2 microscope (Zeiss).

### Electron microscopy

Two P56 adult wild-type and two *SynDIG1* ^β-gal^ homozygous mutant littermates were anesthetized and perfused with ice-cold 0.1 m PBS followed by 4% paraformaldehyde and 2.5% glutaraldehyde in 0.1 m phosphate buffer. The brains were removed, and 80 μm coronal sections were cut with a vibratome (Leica). Sections were dehydrated and flat embedded in Araldite. The hippocampal CA1 regions were identified, and cut and glued to blank resin blocks. Ultrathin 70 nm sections were obtained using a Reichert-Jung Ultramicrotome, thin sections were collected on formvar-coated, single-slot copper grids, stained with uranyl acetate and lead citrate, and examined with a Philips CM-120 electron microscope at 80 kV. Electron microscopic images were acquired using a 2000 × 2000 high-resolution CCD camera (Gatan). Images were processed using DigitalMicrograph software provided by Gatan. Original images were converted to TIFF files and composed in Adobe Photoshop. Regions containing presynaptic vesicles, a synaptic cleft, and a prominent PSD area were counted as synapses. PSD width was measured and quantified with ImageJ (NIH). Perforated synapses were distinguished visually as any two juxtaposed PSDs from one postsynaptic cell drawing from the same pool of synaptic vesicles in the presynaptic terminal. Multiple spine synapses were counted when two apparently unique postsynaptic profiles were seen to be contacted by one presynaptic terminal filled with synaptic vesicles. All quantifications were performed blind to genotype.

### Electrophysiology

Mice were decapitated, and brains put into an ice-cold dissection buffer as follows (in mm): 127 NaCl, 1.9 KCl, 1.2 KH_2_PO_4_, 26 NaHCO_3_, 10 d-glucose, 23 MgSO_4_, and 1.1 CaCl_2_, and saturated with 5% CO_2_ and 95% O_2_ to a final pH of 7.4. The cerebellum was removed, and slices were cut with a vibratome (VT 1000A, Leica) and subsequently maintained in artificial CSF (ACSF) as follows (in mm): 127 NaCl, 26 NaHCO_3_, 1.2 KH_2_PO_4_, 1.9 KCl, 2.2 CaCl_2_, 1 MgSO_4_, and 10 d-glucose, and oxygenated with 95% O_2_ and 5% CO_2_ to a final pH of 7.4 for 1 h at 30°C, and then for up to 5 h at room temperature (RT).

### Field EPSP recordings in hippocampal slices

Slices (400 µm thick) were transferred into a submerged type recording chamber constantly perfused with oxygenated ACSF containing 50 µm bicuculline methobromide (Tocris Bioscience) at 30°C. Field EPSPs (fEPSPs) were recorded in the Schaffer collateral pathway with stimulation and recording electrodes positioned within the stratum radiatum near the CA3 region and in the CA1 region, respectively. fEPSPs were recorded using ACSF-filled glass pipettes (2-3 MΩ), amplified with an Axopatch 2B amplifier (Molecular Devices), digitized at 10 kHz with a Digidata 1320A digitizer (Molecular Devices), and recorded with Clampex 9 software (Molecular Devices). Stimuli (100 µs) were delivered through a concentric bipolar electrode (TM53CCINS, WPI). The same intensity was used during baseline recording (0.067 Hz) and the induction of long-term potentiation (LTP) using 100 stimuli given at 100 Hz (1 s). The baseline was determined by the average of fEPSP initial slopes from the 7 min period immediately before the tetanus. The level of LTP was determined by the average of fEPSP initial slopes from the period between 30 and 60 min after the tetanus. The same slices used for LTP recordings were used to record input–output relation (IOR) for stimulus intensities of 0.1–0.6 mA and paired-pulse facilitation (PPF) for interstimulus intervals of 10, 20, 50, 100, 200, and 500 ms (same stimulation strength as LTP recordings). For each data point, four individual traces were averaged. Data were analyzed and processed using Clampfit 9 and Microsoft Excel. Statistics and visualization were performed with GraphPad Prism. Results between genotypes were statistically compared using one-way ANOVA and Bonferroni’s multiple-comparison test to compare the baseline with LTP for both genotypes as well as LTP between genotypes.

### Whole-cell patch-clamp recording

Slices were transferred into a submerged type recording chamber constantly perfused with oxygenated ACSF supplemented with 50 µm bicuculline methobromide (Tocris Bioscience) at room temperature. Hippocampal pyramidal neurons were visually identified using an Olympus BX50WI upright microscope and an Olympus LumPlanFL 40× water-immersion objective with infrared differential interference contrast through a Hamamatsu C2400 CCD camera. Patch micropipettes (2.5–5 MΩ) were filled with intracellular solution (in mm: 130 CsMeSO_3_, 2.5 CsCl, 7.7 TEA, 4 Mg-ATP, 0.3 Na_2_-GTP, 20 HEPES, 8 NaCl, and 0.2 EGTA, pH 7.2) containing 5 mm QX-314 (Sigma-Aldrich) to prevent action potential firing. All patch-clamp recordings were made in a whole-cell configuration using Clampex 9 to control an Axopatch 200B patch-clamp amplifier (Molecular Devices) through a Digidata 1322A digitizer. Cell capacitance and series resistance were monitored but not compensated for throughout the experiments. A stimulation electrode was positioned in the stratum radiatum. Stimulus intensity was set to evoke 50% EPSC amplitude at a holding potential of −70 mV. Test pulses were given every 15 s. After 5 min of baseline (no more than 8 min after break-in) cells were depolarized to 0 mV. After waiting 15 s for accommodation, 180 pulses were applied with 2 Hz (90 s) before the holding potential was switched back to −70 mV and test pulses resumed after 15 s for 30 min. Signals were sampled at 10 kHz using a 2 kHz low-pass filter. LTP was determined using Clampfit 10 as the average EPSC amplitude recorded 15–30 min after pairing the normalized to the average baseline EPSC. Results between genotypes were statistically compared using one-way ANOVA and Bonferroni’s multiple-comparison test to compare baseline values with LTP values for both genotypes as well as LTP values between genotypes. The AMPA/NMDA ratio was determined by dividing the AMPA portion (measured as the peak amplitude at −70 mV) by the NMDAR portion (current 200 ms after stimulus at +40 mV). For mEPSC recordings, the extracellular ACSF was supplemented with 50 mm sucrose, 1 µm tetrodotoxin (TTX; Tocris Bioscience), and 50 µm bicuculline methobromide (Tocris Bioscience). Cells were held at −70 mV, and miniature events were sampled for up to 30 min at 2 kHz and filtered with a 1 kHz low-pass filter. Events were identified using the inbuilt template-based event detection function in Clampfit. Statistics from 200 randomly selected events per cell were performed with Microsoft Excel and GraphPad Prism.

### Preparation and transfection of organotypic slice cultures

Organotypic hippocampal slice cultures were prepared from P6 to P7 wild-type or *SynDIG1* ^β-gal^ homozygous mutant mice of both sexes, as described previously (Stoppini et al., 1991). Neurons were transfected using particle-mediated gene transfer (130 psi) of enhanced green fluorescent protein (EGFP; Clontech).

### Two-photon imaging and glutamate uncaging

Image stacks (512 × 512 pixels, 1 μm *z*-steps) of two to six secondary and tertiary apical and basal dendritic segments from EGFP-transfected CA1 pyramidal neurons were acquired on a custom two-photon laser-scanning microscope with a pulsed Ti::sapphire laser (930 nm, 0.5–1.5 mW at the sample; SpectraPhysics). Data acquisition was controlled by ScanImage software (Pologruto et al., 2003) written in MATLAB (MathWorks). For all experiments, a single dendrite was imaged. The first time point was acquired in slice culture medium at room temperature, after which the slice was returned to the incubator (35°C). After 1 h, the slice was placed in recirculating ACSF as follows (in mm): 127 NaCl, 25 NaHCO_3_, 1.2 NaH_2_PO_4_, 2.5 KCl, 25 d-glucose, and ∼310 mOsm, pH 7.2, with 2 mm Ca^2+^, 0 mm Mg^2+^, and 1 μm TTX bubbled with carbogen gas (95% O_2_, 5% CO_2_) at 33°C. Following spine identification, 4-methyl-7-nitroindolinyl (MNI)-glutamate (2.5–3.5 mm) was added to the bath and allowed to permeate the slice for 10 min before stimulating the spine. Immediately after stimulation, standard ACSF (2 mm Ca2^+^, 1 mm Mg2^+^) was washed in, and all drugs were washed out.

### Preparation of mouse dissociated hippocampal cultures

Mouse hippocampal neurons from age-matched P0–P2 wild-type or *SynDIG1^β-gal^* homozygous mutant mice of both sexes were dissociated with papain and plated at a density of 1 × 10^5^ cells/well in six-well plates on poly-l-lysine-coated coverslips and maintained in Neurobasal medium supplemented with 1× glutamine, 2% B-27 Supplement, and 100 μg/ml penicillin/streptomycin (all reagents from Invitrogen). At 12 d in vitro (DIV), cultures were treated with TTX (2 μm) or vehicle (DMSO) and incubated for 48 h prior to fixation at 14 DIV and processing for immunocytochemistry. Dissociated hippocampal neurons were fixed in 4% paraformaldehyde in 1× PBS for 10 min at RT. Coverslips were then rinsed in 1× PBS, permeabilized for 10 min at RT with 0.1% Triton X-100 in 1× PBS, and blocked with 5% nonfat milk powder in 1× PBS for 30 min. After incubation with primary antibodies overnight at 4°C and washes in 1× PBS (3 × 10 min), coverslips were incubated with secondary antibodies diluted in blocking solution for 1 h at room temperature. Following washes in 1× PBS (3 × 10 min), coverslips were mounted on microscope slides with Fluoromount-G slide-mounting medium (SouthernBiotech). The following antibodies and dilutions were used: mouse anti-PSD-95 (1:200; NeuroMab); and guinea pig anti-VGluT1 (1:500; Millipore). Single images were acquired using a Zeiss LSM 710 confocal microscope under a 63×/1.4 oil-immersion objective, and with constant settings for gain and offset between groups. Pinhole was set at 1 A.U. and resolution of 1132 × 1132 pixels were used for all images. To quantitatively examine synapse density, confocal images were imported in Zeiss Axiovision 4.4 image analysis software. All channels were thresholded to include all recognizable punctate structures in the analysis. For the calculation of synapse density, a thresholded image of one channel was overlaid as a mask on the second channel. Synapse density was calculated as the percentage of PSD-95 and VGluT1 puncta that overlapped. Data were collected from two independent experiments. Graphs and statistical analyses were plotted and performed in GraphPad Prism software, and data are presented as the mean ± SEM. Statistical significance between experimental and control datasets was assessed by paired Student’s *t* tests. Significance was defined as *p* < 0.05 (*).

## Results

### Characterization of a conditional *SynDIG1^flox^* allele

**Figure 1.**
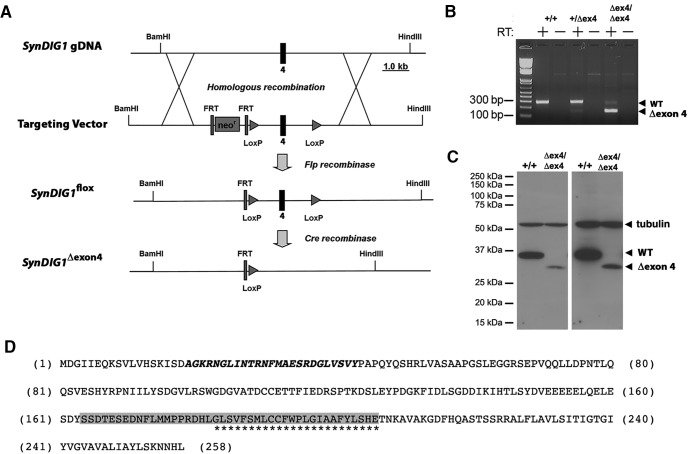
Characterization of a conditional *SynDIG1^flox^* allele. ***A***, Schematic of the conditional *SynDIG1^flox^* allele, which results in the removal of exon 4 (*SynDIG1^Δexon4^*) upon exposure to Cre recombinase. ***B***, RT-PCR using purified tissue RNA from brain tissue of *SynDIG1* wild-type (+/+), heterozygous (+/Δex4), and homozygous (Δex4/Δex4) mutant brains generated by crossing with Nes-cre transgenic mice detects the presence or absence of an mRNA lacking exon 4. A forward primer in exon 3 and a reverse primer in exon 5 produce a 295 or 138 bp product, respectively, in transcripts containing or lacking exon 4. Arrowheads indicate positions of WT and exon 4 lacking mutant products. Note that (+) refers to the unmodified unfloxed *SynDIG1* allele. ***C***, Chemiluminescence immunoblot to detect SynDIG1 expression in brain lysates from wild type (+/+) and homozygous (Δex4/Δex4) mutant animals generated by crossing with CMV-cre transgenic mice detects the presence or absence of SynDIG1 protein lacking amino acids encoded by exon 4. Arrowheads indicate full-length protein (WT), mutant protein (Δexon4), and tubulin (loading control) at 2 min (left) and 4 h (right) exposure times. Note that (+) refers to the unmodified unfloxed *SynDIG1* allele. ***D***, Amino acid sequence of full-length SynDIG1 protein. The epitope recognized by the anti-SynDIG1 antibody is within the region and is shown in bold italic type. Transmembrane amino acids are indicated by asterisks. Amino acids encoded by exon 4 that are eliminated in *SynDIG1^Δexon4^* upon exposure to Cre recombinase are highlighted in gray.

The *SynDIG1* locus is composed of 5 exons. While exons 1 and 2 are noncoding, exons 3 and 4 encode the majority of the protein up to and including the first hydrophobic segment that spans the membrane. Exon 5 encodes the C-terminal 52 aa, including the second hydrophobic segment that does not span the membrane. Transgenic mice with a conditional (floxed) allele of the SynDIG1 gene were obtained from the Mouse Biology Program at the University of California, Davis. This allele (*SynDIG1^flox^*) has two loxP sites surrounding exon 4, which codes for 46 aa residues, including the transmembrane-spanning domain of the SynDIG1 protein (Fig. 1A). However, exons 3 and 5 retain the ability to be spliced in frame and could potentially code for a functional protein with an internal deletion of 46 aa (*SynDIG1^Δexon4^*) upon exposure to Cre recombinase. We investigated this possibility at both the RNA and protein levels. First, we performed RT-PCR on total RNA from brain tissue isolated from homozygous mutant mice (*SynDIG1^Δexon4/Δexon4^*), heterozygous mutant mice (*SynDIG1^Δexon4/+^*), and homozygous wild-type mice (*SynDIG1^+/+^*) generated from crossing with a Nestin-cre driver line. We used primers to amplify the message between exons 3 and 5, and observed an expected 295 bp product in wild-type *SynDIG1^+/+^*and heterozygous *SynDIG1^Δexon4/+^* mice, and a 138 bp product in homozygous *SynDIG1^Δexon4/Δexon4^*mutant mice, which consistent with the presence of SynDIG1 mRNA lacking exon 4 in homozygous mutant mice (Fig. 1B). The presence of a faint 295 bp product in homozygous mutants may be caused by incomplete excision of exon 4 by Cre recombinase. This result suggests that a stable SynDIG1 mRNA lacking exon 4 is produced in *SynDIG1^Δexon4^* mice.

Next, we performed immunoblot analyses to investigate SynDIG1 protein expression in mice in which exon 4 was deleted in all cells generated by crossing to a CMV-cre driver line, creating a homozygous *SynDIG1^Δexon4/Δexon4^* mutant line. Unmodified SynDIG1 protein has a calculated molecular weight of 28.5 kDa, while that for the SynDIG1 protein lacking the 46 aa coded for by exon 4 is calculated to be 23.2 kDa. Immunoblot analysis indicated that *SynDIG1^Δexon4/Δexon4^* mice lacked the full-length SynDIG1 protein with an apparent molecular weight of 30 kDa; however, there was expression of a protein that runs lower than the 37 kDa but higher than the 25 kDa molecular weight marker (Fig. 1C). This smaller band is likely the SynDIG1 protein with the 46 aa internal deletion, which includes the transmembrane domain (Fig. 1D). Given the presence of an expressed mRNA and a protein detectable by our antibody in mutant animals, we could not ignore the potential confounding effects of this gene product in downstream experiments. Hence, we did not use *SynDIG1^Δexon4^* mice in further studies; the remainder of the article describes results using the *SynDIG1* ^β-gal^ mutant allele described below.

### Characterization of SynDIG1 mutant mice

**Figure 2.**
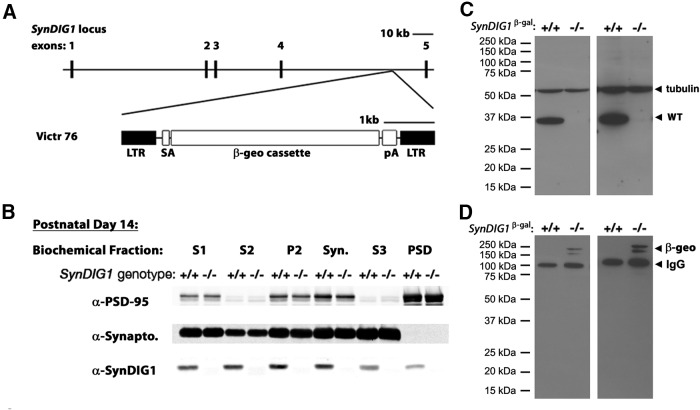
Characterization of *SynDIG1* ^β-gal^ mutant mice. ***A***, Schematic of mouse *SynDIG1* locus showing the insertion site of the β-geo cassette between exon 4 and exon 5 to create *SynDIG1* ^β-gal^ homozygous mutant mice. SynDIG1 expression is disrupted and replaced with β-gal expression. SA, Splice acceptor; pA, synthetic polyA signal/transcriptional blocker; LTR, viral long-terminal repeat segment. ***B***, Immunoblots of biochemical fractions from P14 wild-type (+/+) and *SynDIG1* ^β-gal^ homozygous mutant (−/−) mice using a LI-COR system. Fractions loaded are S1-, S2-, P2-, Syn-, S3-, and PSD-enriched fractions. As a control for the biochemical fractionation, PSD-95 is enriched in the PSD fraction and de-enriched in S2 and S3 while synaptophysin (Synapto) is absent from PSD and enriched in S3. Note that (+) refers to the unmodified *SynDIG1* allele. ***C***, Chemiluminescence immunoblot to detect SynDIG1 expression in brain lysates from wild-type (+/+) and *SynDIG1* ^β-gal^ homozygous mutant (−/−) mice. Arrowheads indicate full-length protein and tubulin (loading control) at 2 min (left) and 4 h (right) exposure times. Note that (+) refers to the unmodified *SynDIG1* allele. ***D***, Chemiluminescence immunoblot to detect β-geo reactivity in brain lysates from WT (+/+) and homozygous (−/−) mutant mice. Arrowheads indicate bands for β-geo and mouse IgG (recognized by secondary antibody) at 2 min (left) and 4 h (right) exposure times. Note that (+) refers to the unmodified *SynDIG1* allele.

To determine the function of SynDIG1 *in vivo*, we acquired a commercially available *SynDIG1* mutant mouse line from the TIGM. The targeting strategy used uses the Victr 76 viral vector (Hansen et al., 2008) to insert a cassette encoding a β-geo fusion protein (β-galactosidase-Neo^R^) in between exons 4 and 5 of the *SynDIG1* locus to disrupt protein expression such that β-gal functions as a reporter driven by the *SynDIG1* promoter (Fig. 2A). Normal SynDIG1 expression should be greatly reduced or completely ablated due to the presence of a strong splice acceptor and transcriptional blocker (Fig. 2A). These mice are referred to as *SynDIG1* ^β-gal^ mutants. We did not observe any obvious phenotypic differences between mutant mice and WT littermates, and litters showed expected Mendelian ratios in sex and genotype.

Because *SynDIG1* ^β-gal^ mice still retain the *SynDIG1* locus, biochemical fractionation followed by immunoblot analyses was performed on brain tissue from WT and homozygous mutant animals to check for retained protein expression (Fig. 2B). In P14 WT mice, SynDIG1 is present in S1-, S2-, P2-, Syn-, S3-, and PSD-enriched biochemical fractions (Fig. 2B). To ensure the enrichment of biochemical fractions, levels of PSD-95 and synaptophysin were measured. The postsynaptic protein PSD-95 is diminished in the S3 fraction and greatly enriched in the PSD fraction. The presynaptic protein synaptophysin is enriched in the Syn and S3 fractions, whereas it is undetected in the PSD fraction. When using the less sensitive but more quantifiable LI-COR-based method, *SynDIG1* ^β-gal^ homozygous mutant brains do not show any evident SynDIG1 expression in any fraction at P14, although the trapped allele could potentially result in a SynDIG1 protein fragment with a calculated molecular weight of 23.6 kDa recognized by the SynDIG1 antibody. Using the more sensitive peroxidase-based chemiluminesence, there is a faint band detected in the *SynDIG1* ^β-gal^ homozygous mutant lysates upon long-term film overexposure (Fig. 2C); however, this band is not at the appropriate molecular weight range of the predicted SynDIG1 protein fragment. One possibility is that the *SynDIG1* ^β-gal^ allele is a hypomorph of SynDIG1 expression and that a small contingent of functional SynDIG1 protein remains in *SynDIG1* ^β-gal^ homozygous mutant mice. However, because the resulting SynDIG1 trapped allele is predicted to generate a much smaller protein, it is equally possible that this band represents a nonspecific protein recognized by the SynDIG1 antibody. The trapped allele does not appear to generate a fusion protein between SynDIG1 and the β-geo cassette because immunoblotting with anti-β-gal antibodies revealed a signal that is not detected by the SynDIG1 antibody (Fig. 2D).

### Analyses of the β-gal reporter expression in developing mouse brain

**Figure 3.**
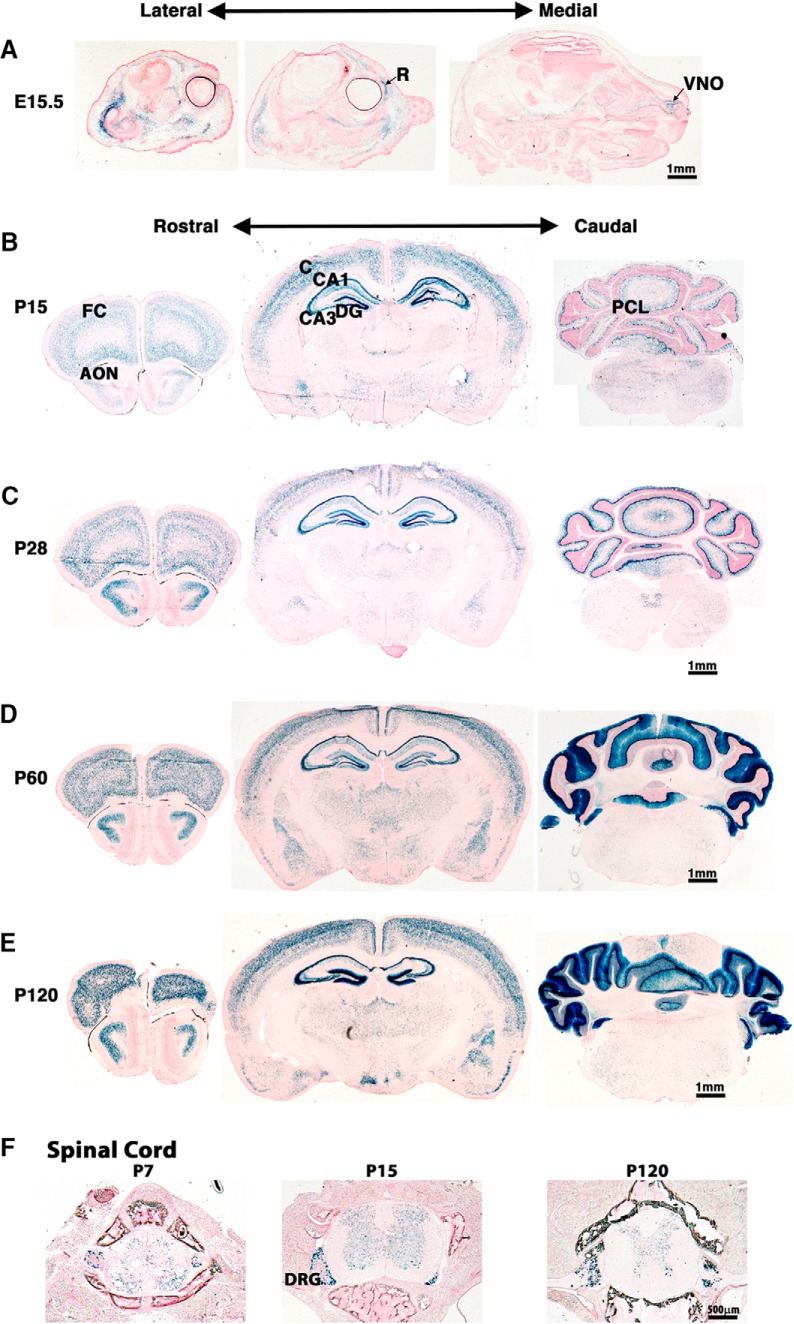
Expression profile of SynDIG1 reporter in the developing mouse brain. ***A***, Sagittal sections from the entire head of E15.5 *SynDIG1* ^β-gal^ homozygous mutant mouse show β-gal reporter activity (blue) within the retina (R) and vomeronasal organ (VNO), but little β-gal activity in the brain. ***B–E***, Coronal sections of P15 (***B***), P28 (***C***), P60 (***D***), and P120 (***E***) mutant mouse brains show β-gal expression in the CA1, CA3, and DG in the hippocampus; in the PCL of the cerebellum; in the frontal cortex (FC); in the AON; and in the cortex (C). Scale bars, 1 mm. ***F***, Cross sections of P7, P15, and P120 vertebrae show β-gal expression in the dorsal root ganglia (DRG) and within the spinal cord. Scale bar, 500 nm. All sections are counterstained with nuclear fast red.

*In situ* hybridization with antisense probes previously showed that SynDIG1 transcript is present within the Purkinje cell layer (PCL) of the cerebellum and throughout the hippocampus at P20 (Kalashnikova et al., 2010), and the SynDIG1 profile in the Allen Mouse Brain Atlas indicates strong expression in cerebellum (http://mouse.brain-map.org/experiment/show/70723307; http://mouse.brain-map.org/experiment/show/73931426; Lein et al., 2007). The β-gal reporter activity was investigated in cryosections from *SynDIG1* ^β-gal^ homozygous mutant mice stained to label functional β-gal expression. The *SynDIG1* promoter is active at E15.5 within the retina and vomeronasal organ (Fig. 3A). Little β-gal expression is evident within the brain until after birth. At P15 and P28, there is β-gal expression in the cerebral cortex, anterior olfactory nucleus (AON), PCL of the cerebellum, and throughout the hippocampus, including the CA1, CA3, and dentate gyrus (DG) regions (Fig. 3B,*C*). The expression of β-gal appears to increase substantially within the PCL of the cerebellum at P60 and P120 (Fig. 3D,*E*). Intriguingly, β-gal expression is not uniform throughout the cortical layers from P7 and continuing to P120 (Fig. 3B*–E*). Punctate β-gal expression was also found in the gray matter portions of the spinal cord and within dorsal root ganglia at ages P7, P15, and P120 (Fig. 3F).

### SynDIG1-deficient synapses are structurally immature

**Figure 4.**
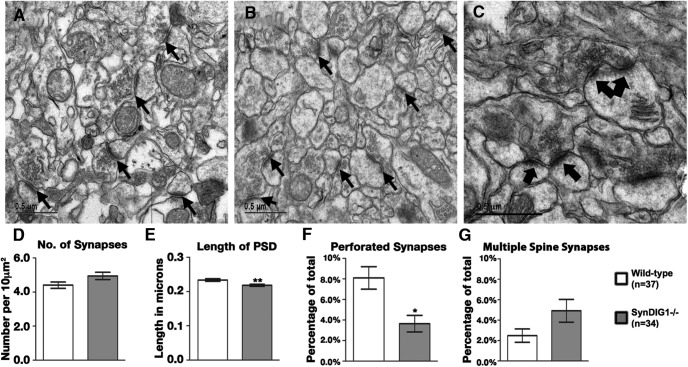
Synapse ultrastructure is altered in *SynDIG1* mutant mice. ***A***, ***B***, Representative electron micrographs from the CA1 region of the hippocampus from P56 WT (***A***) and *SynDIG1* ^β-gal^ homozygous mutant littermates (***B***). Example synapses are indicated by black arrows. ***C***, Higher-magnification image from WT mouse hippocampus illustrating a stereotypical perforated synapse (conjoined black arrows) and multiple spine synapses (two separated arrows). Scale bar, 0.5 µm. ***D–G***, Quantification of multiple images (WT, *n* = 37; *SynDIG1* ^β-gal^ homozygous mutant, *n* = 34) reveal a trend toward increased synapse number (WT, 4.40 synapses/10 µm^2^; mutant, 4.94 synapses/10 μm^2^; *p* = 0.057; ***D***), a statistically significant decrease in average PSD length (WT, 0.233 µm; *SynDIG1* ^β-gal^ homozygous mutant, 0.218 µm; *p* = 0.016; ***E***), a statistically significant decrease in the number of perforated synapses (WT, 8.10% perforated synapses; *SynDIG1* ^β-gal^ homozygous mutant, 3.65% perforated synapses; *p* = 0.002; ***F***), and a trend toward an increased number of multiple spine synapses (WT, 2.49% multiple spine synapses; *SynDIG1* ^β-gal^ homozygous mutant, 4.92% multiple spine synapses; *p* = 0.059; ***G***) in *SynDIG1* ^β-gal^ homozygous mutant mice compared with WT. Error bars represent the SEM.

To investigate structural changes in excitatory synapses, the CA1 region of the hippocampus was analyzed in two 2-month-old *SynDIG1* ^β-gal^ homozygous mutant mice and two WT littermates with electron microscopy (Fig. 4A*–C*). Synapse ultrastructure in multiple electron micrographs was determined by several criteria, including the number of asymmetrical synapses per unit area, the length of the PSD in each synapse, and the percentage of perforated and multiple spine synapses. Perforated synapses are defined by a discontinuous PSD and reflect mature synapses that have undergone structural changes in response to activity and contain a higher number of AMPA receptors compared with classic synapses (Ganeshina et al., 2004a,b). Multiple spine synapses occur when two PSDs from two unique postsynaptic cells draw from one pool of presynaptic vesicles derived from one presynaptic cell. Quantification of multiple images revealed a trend toward an increase in excitatory synapse number in mutant compared with WT mice (*p* = 0.057; Fig. 4D). There is a statistically significant decrease in average PSD length in excitatory synapses of *SynDIG1* ^β-gal^ homozygous mutant mice (Fig. 4E). Moreover, we also found a statistically significant decrease in the number of perforated synapses in *SynDIG1* ^β-gal^ homozygous mutant mice (Fig. 4F) and a trend toward an increase in the number of multiple spine synapses in *SynDIG1* ^β-gal^ mutant mice compared with WT mice (*p* = 0.059; Fig. 4G). These data are consistent with an overall decrease in synapse maturity in *SynDIG1* ^β-gal^ homozygous mutant mice.

### SynDIG1-deficient synapses are functionally immature

**Figure 5.**
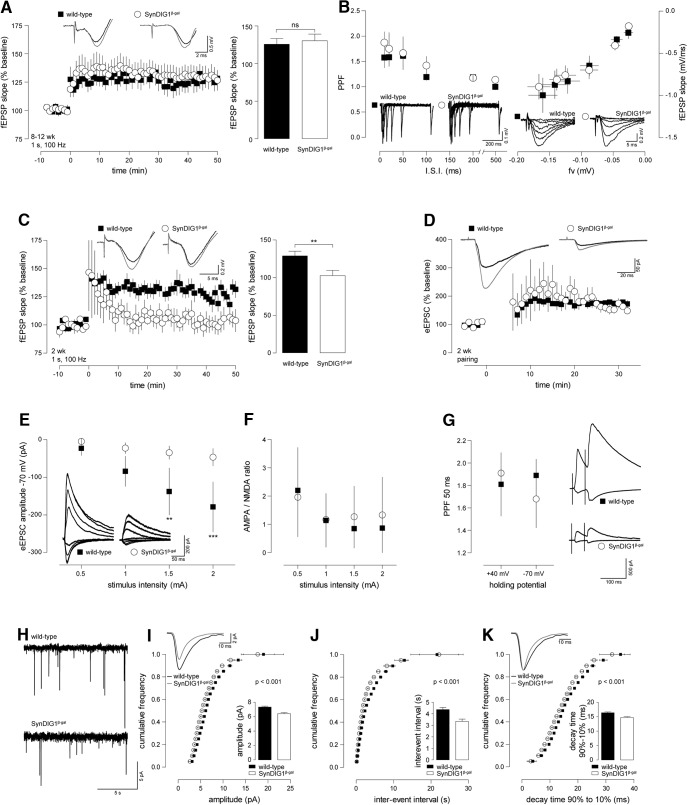
Synaptic transmission and plasticity is disturbed in 2-week-old but not in adult *SynDIG1* ^β-gal^ mutant mice. Acute brain slices from *SynDIG1* ^β-gal^ homozygous mutant (○, *SynDIG1* ^β-gal^) and litter-matched WT controls (▪) were used to record Schaffer collateral LTP and whole-cell patch-clamp experiments on CA1 pyramidal cells. ***A***, fEPSP recorded in slices from 8- to 12-week-old mice. *SynDIG1* ^β-gal^ homozygous mutant and WT controls showed significant LTP after a 1 s 100 Hz tetanic stimulation. The level of potentiation was not different between the genotypes (WT: baseline, 99.6 ± 0.8%; LTP, 125.6 ± 7.6%; *p* < 0.05 vs baseline; *n* = 12; *SynDIG1* ^β-gal^ homozygous mutant: baseline, 100.5 ± 0.4%; LTP, 130.2 ± 8.9%; *p* < 0.01 vs baseline; *n* = 13). Insets at top show traces from representative recordings before and after (gray traces) tetanization. The left panel shows averaged time courses of all experiments with traces from representative recordings on top. Statistics are illustrated in the bar diagram on the right. ***B***, *SynDIG1* ^β-gal^ homozygous mutant mice displayed normal PPF (left) and IOR (right) when compared with WT animals. Insets beneath data points show representative recordings. ***C***, fEPSP recorded from 2-week-old mice. A 1 s 100 Hz tetanus leads to potentiation of fEPSP in WT [baseline, 99.9 ± 0.9%; LTP, 128.3 ± 6.7%; *p* < 0.001 (WT baseline vs LTP); *n* = 10] but not *SynDIG1* ^β-gal^ homozygous mutant mice [baseline, 99.4 ± 0.5%; LTP, 102.6 ± 7.0%; *p* < 0.01 (*SynDIG1* ^β-gal^ homozygous mutant vs WT LTP); *n* = 6]. The left panel shows the averaged time courses of all experiments with traces from representative recordings at the top. Statistics are illustrated in the bar diagram on the right. ***D***, Pairing-induced LTP of evoked EPSC (eEPSC) in hippocampal slices of 2-week-old animals was normal in *SynDIG1^β-gal^* (baseline, 99.4 ± 0.6%; LTP, 168.5 ± 15.4%; *p* < 0.001 vs baseline; *n* = 3) compared with WT mice (baseline, 100.1 ± 0.04%; LTP, 175.7 ± 10.6%; *p* < 0.001 vs baseline; *n* = 4). Insets at the top show traces from representative recordings before and after (gray traces) pairing. ***E***, EPSCs were evoked by increasing stimulus intensities recorded at holding potentials of −70 and +40 mV. At a holding potential of −40 mV, eEPSC amplitudes were significantly higher in WT mice than in *SynDIG1^β-gal^* mutants [stimulus intensity (si) = 0.5 mA, −22.8 ± 19.3 pA; si = 1 mA, −84.2 ± 40.1 pA; si = 1.5 mA, −137.9 ± 62.4 pA; si = 2 mA, −179.8 ± 66.6 pA; *n* = 9; *SynDIG1^β-gal^*; si = 0.5 mA, −4.7 ± 1.2 pA; si = 1 mA, −22.5 ± 15.6 pA; si = 1.5 mA, −34.6 ± 17.6 pA; si = 2 mA, −46.6 ± 23.1 pA; *n* = 4; two-way ANOVA and Bonferroni’s post-test: *F*_(1,44)_ = 35.4; 1.5 pA, *p* < 0.01; 2 pA, *p* < 0.001]. Insets at the bottom show traces from representative recordings. ***F***, The AMPAR/NMDAR ratio did not differ between WT and *SynDIG1^β-gal^* in the recordings from ***E*** for all stimulus intensities. ***G***, No difference was found in PPF (50 ms interstimulus interval) between WT and *SynDIG1^β-gal^*. Insets on the right show traces from representative recordings. ***H***, Sample recordings of mEPSCs recorded from CA1 pyramidal neurons in acute slices of 2-week-old WT and *SynDIG1* ^β-gal^ homozygous mutant mice. ***I–K***, Cumulative histograms show significant reduction in amplitude (***I***), interevent interval (***J***), and decay time (***K***) of mEPSC recorded in *SynDIG1* ^β-gal^ homozygous mutant compared with WT mice (bin size, 0.5; *t* tests for each binned data point). Inset at top contains traces averaged from all mEPSCs of one representative experiment for each genotype drawn to scale (***I***) and normalized to peak (***K***). Averages are shown in bar diagrams at the bottom (amplitude: WT, 7.3 ± 0.1 pA, *n* = 8; *SynDIG1* ^β-gal^ homozygous mutant, 6.4 ± 0.1 pA, *n* = 6. Interevent interval: WT, 4.3 ± 0.2 s; *SynDIG1* ^β-gal^ homozygous mutant, 3.3 ± 0.2 ms. Decay time: WT, 16.4 ± 0.3 ms; *SynDIG1* ^β-gal^ homozygous mutant, 14.7 ± 0.3 ms).

To determine the functionality of *SynDIG1* ^β-gal^ synapses, we performed fEPSP recordings in hippocampal slices of 8- to 12-week-old *SynDIG1* ^β-gal^ mutants. LTP was elicited in the presence of the GABA_A_ receptor antagonist bicuculline by a weak tetanic stimulation (1 s, 100 Hz) in order to distinguish subtle changes in synaptic plasticity that might remain concealed when stronger stimulus paradigms are used. Using 8- to 12-week-old animals, we found no alterations in the magnitude of LTP expressed by *SynDIG1* ^β-gal^ mutants compared with WT controls (Fig. 5A). In slices from the same animals, the probability of presynaptic transmitter release, as monitored by PPF, was comparable between the genotypes (Fig. 5B). Additionally, we found no changes in the IOR of the fEPSP strength in response to increasing stimulus intensities between mutants and controls (Fig. 5B). Remarkably, when using the same weak (1 s, 100 Hz) LTP paradigm in 2-week-old animals, we found a complete lack of LTP in *SynDIG1* ^β-gal^ homozygous mutant mice in contrast to WT controls (Fig. 5C). To evaluate whether this effect is due to disturbed synaptic plasticity or a deficit in synaptic transmission, we performed whole-cell patch-clamp experiments on CA1 pyramidal neurons of *SynDIG1^β-gal^* mice.

First, we looked at pairing-induced LTP. In this experiment, cells are held at 0 mV to release the Mg^2+^ block of the NMDA receptor. In this configuration, the stimulation of presynaptic terminals at 2 Hz for 90 s readily leads to postsynaptic potentiation of the EPSC triggered by Ca^2+^ influx through NMDA receptors. *SynDIG1^β-gal^* exhibited the same amount of pairing-induced LTP as did WT controls (Fig. 5D), arguing that SynDIG1^β-gal^ mice do not lack the mechanisms for synaptic plasticity downstream of the NMDA receptor. What became immediately apparent, however, was that EPSC amplitudes of 2-week-old *SynDIG1^β-gal^* mice are significantly smaller than those of WT controls, while the AMPA/NMDA ratio is unchanged (Fig. 5E,*F*). Additionally, we were unable to identify any defect in presynaptic function as we could observe normal PPF (Fig. 5G). These observations are consistent with a model where SynDIG1-deficient synapses are less mature and show a reduced density of AMPA- and NMDA-type glutamate receptors. The remaining channels are sufficient to trigger synaptic plasticity upon NMDA receptor activation; however, the reduced amount of AMPA receptor present in the postsynapse is insufficient to depolarize the postsynaptic cell enough to release the Mg^2+^ block from the NMDA receptor. Therefore, the weak 1 s 100 Hz tetanus did not elicit LTP, while pairing-induced LTP was not affected as there the Mg^2+^ block is released by depolarization.

To evaluate potential changes in postsynaptic AMPA receptor function more carefully, we monitored mEPSC by whole-cell patch-clamp recordings (Fig. 5H, representative recordings). Accordingly, mEPSC amplitude in *SynDIG1* ^β-gal^ homozygous mutant CA1 pyramidal neurons was significantly reduced compared with WT controls (Fig. 5I). At the same time, we found an increase in mEPSC frequency (Fig. 5J, visualized as the interevent interval, the inverse of frequency), possibly due to mechanisms compensating for the loss in AMPA receptor density. Additionally, mEPSC of *SynDIG1^β-gal^* homozygous mutant mice display a significantly faster decay time than do WT controls (Fig. 5K). Faster EPSC decay additionally reduces the amount of current conducted by the AMPA receptor, which might contribute to the reduced depolarization of postsynaptic cells.

### SynDIG1-deficient synapses exhibit increased short-term structural plasticity

**Figure 6.**
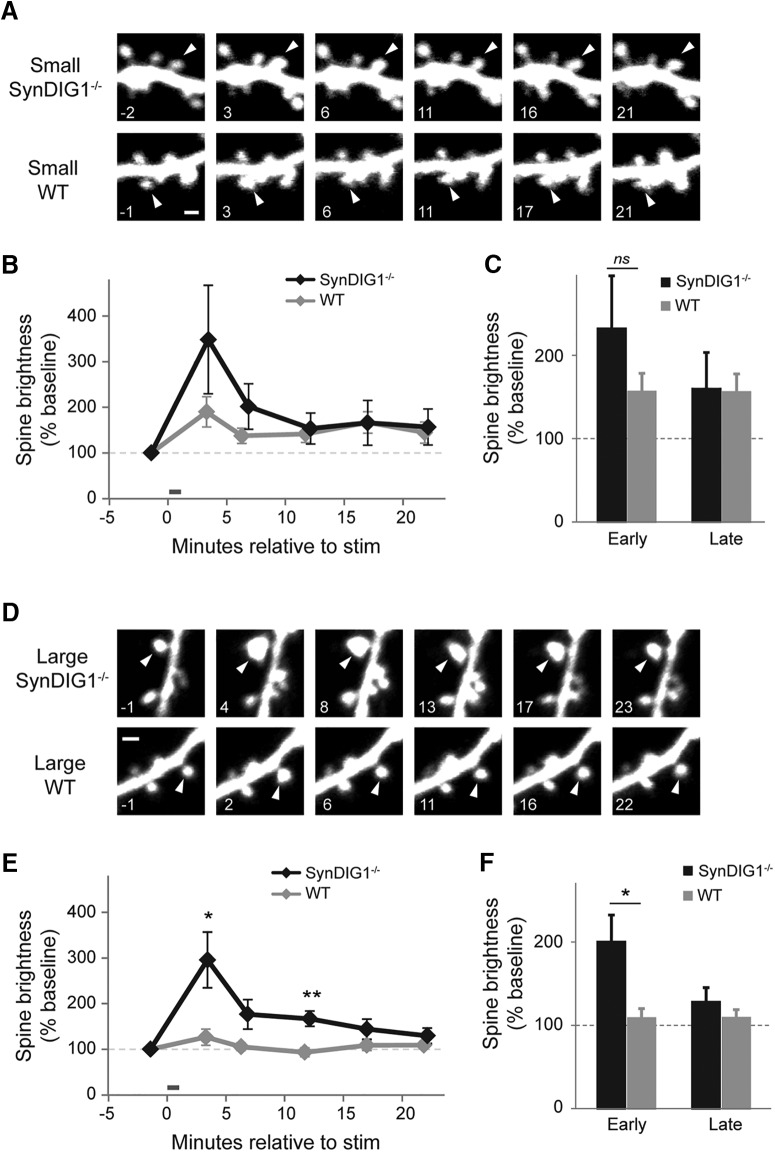
SynDIG1 limits early-phase structural plasticity in large spines. ***A***, Representative images illustrating small stimulated spines (arrowheads) on EGFP-expressing neurons from *SynDIG1* ^β-gal^ homozygous mutant neurons (top) and neurons from WT mice (bottom). Time stamps are in minutes relative to LTP stimulation. Small spines are defined as <85% of average neighbor brightness. Scale bar, 1 µm. ***B***, Time course of small spine structural plasticity. There are no significance differences between genotypes (WT, *n* = 14 stimulated target spines on 14 cells; *SynDIG1* ^β-gal^ homozygous mutant, *n* = 13 stimulated target spines on 13 cells; *p* > 0.2 for all comparisons). Error bars indicate ±SEM. ***C***, The average early (<15 min after stimulation) and late (>15 min after stimulation) change in small spine brightness by genotype. There are no significant differences between genotypes (*p* > 0.4 for all comparisons). Error bars represent ±SEM. ***D***, Representative images illustrating large stimulated target spines (arrowheads) on EGFP-expressing neurons from *SynDIG1* ^β-gal^ homozygous mutant neurons (top) and WT neurons (bottom). Time stamps are in minutes relative to LTP stimulation. Large spines are defined as >85% of average neighbor brightness. Scale bar, 1 µm. ***E***, Time course of large spine structural plasticity. Large stimulated spines on WT neurons did not enlarge after stimulation (black line; *n* = 8 spines on 8 cells). Large stimulated spines on *SynDIG1* ^β-gal^ homozygous mutant neurons enlarged significantly 3 and 12 min after stimulation (gray line; *n* = 7 spines on 7 cells; *p* < 0.05). Error bars represent the SEM. ***F***, The average early (<15 min after stimulation) and late (>15 min after stimulation) change in large spine brightness by genotype. Large stimulated spines on *SynDIG1* ^β-gal^ homozygous mutant neurons enlarged significantly more than those of WT neurons during early-phase, but not late-phase, structural plasticity (*p* < 0.05). Error bars represent ±SEM.

Given the changes in excitatory synapse structure and function, we investigated whether there were changes in other aspects of synapse development. Specifically, we observed structural remodeling upon two-photon photolysis of caged glutamate (MNI-glutamate) at single spines of hippocampal CA1 pyramidal neurons (Fig. 6). Hippocampal slice cultures from P6 to P7 WT and *SynDIG1* ^β-gal^ homozygous mutant mice were transfected with EGFP to visualize spines on dendritic segments from CA1 pyramidal neurons using a two-photon laser-scanning microscope. Stimulation with a protocol that mimics LTP results in long-lasting increased spine volume, as measured by EGFP fluorescence that is dependent on original spine volume (Matsuzaki et al., 2004). Small spines (defined as spines that are <85% of the average neighbor brightness) consistently exhibit increased long-lasting enlargement of spine volume upon LTP stimulation in contrast to large spines (defined as spines that are >85% of the average neighbor brightness; Matsuzaki et al., 2004). Small spines from WT animals exhibited an initial twofold increased spine volume upon LTP stimulation compared with baseline that remains statistically different at later time points (Fig. 6A*–C*), whereas large WT spines did not respond to LTP stimulation at any time point (Fig. 6D*–F*). Interestingly, small spines from *SynDIG1* ^β-gal^ homozygous mutant animals have a trend toward increased initial spine brightness upon LTP stimulation (*p* = 0.07; Fig. 6A*–C*). Strikingly, large spines from *SynDIG1* ^β-gal^ homozygous mutants exhibit a significant threefold short-term increase in spine brightness compared with baseline in contrast to large spines from WT animals (*p* = 0.01; Fig. 6D*–F*).

### Synapse composition of SynDIG1-deficient synapses is unchanged

**Figure 7.**
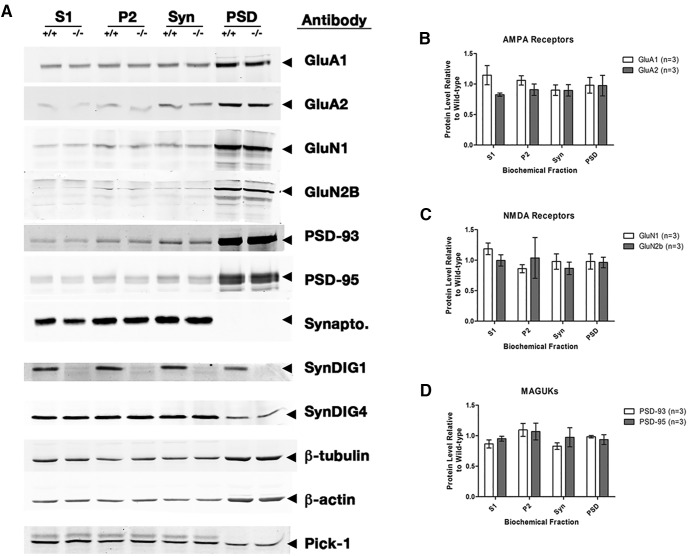
Synapse composition is unaltered in SynDIG1-deficient synapses. ***A***, Representative immunoblots of biochemical fractions isolated from WT and *SynDIG1* ^β-gal^ homozygous mutant P14 mouse brain tissue showing levels of GluA1, GluA2, GluN1, GluN2B, PSD-93, PSD-95, synaptophysin (Synapto), SynDIG1, SynDIG4, and PICK-1 present in the S1-, P2-, Syn-, and PSD-enriched fractions. Loading controls are provided by β-actin and β-tubulin immunoreactivity. ***B–D***, Graphs depict the ratio of *SynDIG1* ^β-gal^ homozygous mutant protein relative to WT levels of AMPA receptor subunits (***B***), NMDA receptor subunits (***C***), and PSD-93 and PSD-95 (***D***) in the PSD-enriched fractions. Data are the average of three independent biochemical fractionation experiments; each experiment used four to six mouse brains of each genotype. Error bars represent ±SEM.

Because of the significant changes that we found in the structure and function of excitatory synapses, we investigated whether protein levels of candidate AMPA and NMDA receptor subunits and MAGUK scaffolding proteins PSD-93 and PSD-95 were altered in SynDIG1-deficient synapses. Protein was isolated from P13–P15 WT and *SynDIG1* ^β-gal^ homozygous mutant mouse forebrains in three independent biochemical fractionation experiments. We tested for changes in protein levels between WT and mutant samples in the following four fractions: postnuclear, membrane enriched, synaptosomal enriched, and postsynaptic density enriched (Fig. 7). These levels were compared with the cytoskeletal proteins β-actin and β-tubulin, which are present in all fractions. SynDIG1 is greatly diminished in all fractions from mutant tissue at this age. The SynDIG1 ortholog SynDIG4 [also known as Prrt1 (proline-rich transmembrane protein 1)] is also present in all fractions, and remains unchanged between WT and mutant samples, as is the scaffolding protein PICK-1 (protein kinase C binding protein 1; Fig. 7A). Proteins from each fraction were normalized to β-tubulin or β-actin, and the ratio to WT levels was determined. There is no statistically significant difference in protein levels between WT and mutant mice in the AMPA receptor subunits GluA1 and GluA2 (Fig. 7B), the NMDA receptor subunits GluN1 and GluN2A (Fig. 7C), or the MAGUKs PSD-93 and PSD-95 (Fig. 7D). While there may be minor trends of difference between WT and mutant protein levels in some biochemical fractions, the PSD fractions show similar protein levels of all tested candidate proteins.

### Activity-dependent synapse development is impaired in SynDIG1-deficient neurons

**Figure 8.**
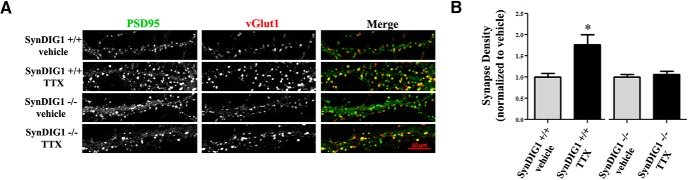
Activity-dependent synapse development was abolished upon the loss of SynDIG1. ***A***, Loss of SynDIG1 inhibits activity-dependent excitatory synapse development. Immunostaining of PSD-95 (green) and presynaptic VGluT1 (red) proteins is shown. Dissociated hippocampal neurons from *SynDIG1* ^β-gal^ homozygous mutant (−/−) or WT (+/+) mice were treated with vehicle (DMSO) or TTX (2 μm) at 12 DIV, fixed, and stained at 14 DIV. Synapses are defined as the overlap of presynaptic and postsynaptic clusters. Scale bar, 10 μm. ***B***, Graph depicts the density of PSD-95/VGluT1 colocalized puncta. Normalized values relative to untreated cells are shown for the average of two independent experiments (*n* = 20-30 cells/condition). Error bars represent ±SEM. *p < 0.05.

SynDIG1 localization at excitatory synapses is sensitive to activity level in hippocampal neurons (Kalashnikova et al., 2010). To test whether SynDIG1 deficiency resulted in deficits in activity-dependent synapse development, dissociated cultures of hippocampal neurons from WT and mutant mice were prepared. At 12 DIV, cultures were treated with 2 µm TTX or vehicle and fixed at 14 DIV for immunocytochemistry to label synapses with antibodies against VGluT1 and PSD-95 as presynaptic and postsynaptic markers, respectively (Fig. 8A). Quantification of multiple images from two independent experiments revealed increased synapse density (measured as the overlap of VGluT1 and PSD-95 puncta) in WT neurons treated with TTX compared with vehicle-treated cultures, while synapse density failed to increase upon TTX treatment in *SynDIG1* ^β-gal^ homozygous mutant neurons compared with vehicle-treated cultures (Fig. 8B), indicating that activity-dependent synapse development requires SynDIG1.

## Discussion

It proved to be unfortunate that the putative conditional null *SynDIG1^Δexon4^* mutant mouse line had a retained protein product. As such, we did not use that line for any phenotypic analyses and recommend that other researchers instead use the *SynDIG1* ^β-gal^ mutant line that we characterized for all subsequent experiments. The secondary function of this line as a reporter also proved quite useful in determining when and where SynDIG1 might be expressed. The SynDIG1 profile in the Allen Mouse Brain Atlas (http://mouse.brain-map.org/experiment/show/70723307; http://mouse.brain-map.org/experiment/show/73931426) matches the expression pattern observed here in the PCL of the cerebellum. Our data further suggest SynDIG1 expression throughout the developing and adult hippocampus and cortex, which is consistent with published *in situ* hybridization studies (Kalashnikova et al., 2010). The SynDIG1 profile in the Allen Mouse Brain Atlas does not indicate strong SynDIG1 transcription elsewhere in the brain, suggesting that the reporter line is a more sensitive representation of SynDIG1 expression. While the presence of β-galactosidase shows only promoter activity, our immunoblots from forebrain tissue indicate that SynDIG1 is indeed expressed outside of the cerebellum. Furthermore, the β-gal expression also matches immunoblot results from a recent publication comparing SynDIG1 expression and the related protein SynDIG4/Prrt1 in rat brain (Kirk et al., 2016).

Together, the results presented here support the conclusion that SynDIG1-deficient excitatory synapses are impaired in their structural and functional properties, and suggest that these synapses are of an immature phenotype based on multiple independent lines of evidence. First, *SynDIG1* ^β-gal^ homozygous mutant mice have a significant decrease in PSD length, suggesting a reduction in excitatory synapse maturation. As a synapse matures and strengthens, it will recruit more synaptic proteins, increasing the size of the PSD (Zheng et al., 2011). In support of this interpretation, the number of perforated synapses is decreased significantly in *SynDIG1* ^β-gal^ homozygous mutant mice compared with WT animals. Perforated synapses are thought to be indicative of synaptic remodeling during plasticity that occurs normally during development (Calverley and Jones, 1990) and are more likely to contain AMPA receptors than nonperforated synapses (Ganeshina et al., 2004a,b). Furthermore, there is usually an increase in perforated synapses following LTP induction (Toni et al., 2001). Therefore, a reduction of perforated synapses in *SynDIG1* ^β-gal^ homozygous mutant mice also supports the idea that these excitatory synapses are weaker and less mature on average than their WT littermates. The trend toward more synapses in *SynDIG1* ^β-gal^ homozygous mutant mice may also be linked to the decreased PSD length and perforated synapse number. It is possible that this trend reflects a compensatory mechanism trying to overcome a lack of mature synapses. Such a compensatory mechanism might also explain the trend toward increased multiple spine synapses observed in *SynDIG1* ^β-gal^ homozygous mutant mice compared with WT animals if there is an increase in the drive to form synapses, so more postsynaptic target cells will respond to a presynaptic stimulus. Overall, the loss of SynDIG1 appears to fundamentally alter the structural properties of the normal maturation process of excitatory synapse development.

Second, decreased mEPSC amplitudes in *SynDIG1* ^β-gal^ homozygous mutant hippocampal slices suggest a decreased responsiveness of postsynaptic cells to glutamate release, another hallmark of smaller, less mature synapses. Moreover, the significant decrease in the interevent interval of mEPSC events in *SynDIG1* ^β-gal^ homozygous mutant slices indicates that there are more synapses compared with WT mice. These data corroborate the changes observed in the ultrastructure of the excitatory synapses. Namely, there are more yet weaker synapses present in *SynDIG1* ^β-gal^ homozygous mutant mice.

Third, high-frequency glutamatergic stimulation has been shown to increase the volume and response properties of individual spines. This event is correlated with the recruitment of postsynaptic scaffolding molecules and is thought to indicate an increase in spine maturity (Bosch et al., 2014; Colgan and Yasuda, 2014; Meyer et al., 2014). Small spines exhibit increased long-lasting enlargement of spine volume upon LTP stimulation in contrast to large spines (Matsuzaki et al., 2004). Large spines are thought to represent “memory” synapses that are stable and fixed, while small spines are “learning” synapses that are substrates for structural remodeling. Indeed, new spines subjected to LTP stimulation are long lasting, indicating that small new spines can mature into persistent larger spines (Hill and Zito, 2013). Small spines from both WT and mutant animals exhibit increased spine volume compared with baseline. In contrast, large spines from mutant neurons showed significantly increased spine volume compared with large spines from WT neurons. Thus, SynDIG1-deficient spines exhibit hyper-reactive enlargement upon glutamate uncaging compared with WT spines.

It remains unclear what mechanism is responsible for this excessive increase in volume in *SynDIG1* ^β-gal^ homozygous mutant mice upon glutamate stimulation. One might expect that hyper-reactive spine enlargement, if long lasting, would result in an increased number of mature synapses. However, we observed the opposite results in our ultrastructure and electrophysiology experiments. We propose that the loss of SynDIG1 impairs the ability of excitatory synapses to mature fully. Spine stimulation via uncaging of glutamate could trigger a maturation response that was primed to be initiated, but fails to do so without SynDIG1. This interpretation might explain why the *SynDIG1* ^β-gal^ homozygous mutant spines return to WT levels so soon after glutamate uncaging. Moreover, this “primed yet unable to mature” model also explains why there is a dearth of mature synapses in *SynDIG1* ^β-gal^ homozygous mutant mice; without SynDIG1, normal spine maturation is significantly reduced.

How does SynDIG1 influence excitatory synapse maturation? The biochemical fractionation data suggest that the loss of SynDIG1 does not drastically alter the overall composition of excitatory synapses. Preliminary biotinylation studies indicate that surface glutamate receptors are also unchanged (our unpublished observations). However, it remains possible that a yet to be determined upstream or downstream interacting partner of SynDIG1 is drastically altered in *SynDIG1* ^β-gal^ homozygous mutants. Interestingly, a recent publication (Kaur et al., 2016) demonstrated that palmitoylation of SynDIG1 at two conserved Cys residues in an activity-dependent manner regulates its subcellular localization and function. Therefore, it is tempting to speculate that activity-dependent palmitoylation of SynDIG1 may underlie its role in activity-dependent synapse development that is demonstrated here.

In a dissociated culture of rat hippocampal neurons SynDIG1 overexpression leads to an increase in the number and strength of excitatory synapses, and SynDIG1 knockdown results in decreased number and strength of excitatory synapses (Kalashnikova et al., 2010), indicating that the level of SynDIG1 is intimately related to the number and strength of excitatory synapses. We found that a reduction in synapse strength was phenocopied in *SynDIG1* ^β-gal^ homozygous mutant hippocampi; however, the effect of genetic removal of SynDIG1 *in vivo* on synapse number is not consistent. It is possible that the SynDIG1-deficient (weaker) synapses that fail to mature are not eliminated, thereby leading to an overall increase in synapse number. This interpretation suggests that synapse formation per se is unaffected in SynDIG1-deficient animals. A recent study (Lovero et al., 2013) in rat hippocampal slice culture concluded that SynDIG1 functions primarily as a synaptogenic factor to regulate excitatory synapse number. This study showed that knockdown of SynDIG1 in a hippocampal slice culture resulted in decreased EPSC without altering AMPA/NMDA ratio or presynaptic release (Lovero et al., 2013). However, in contrast to the *in vivo* studies presented here as well as to published studies in dissociated rat hippocampal neurons (Kalashnikova et al., 2010), SynDIG1 knockdown in a hippocampal slice culture resulted in decreased mEPSC frequency, with a trend toward decreased mEPSC amplitude (Lovero et al., 2013). The source of the discrepancy could be due to species differences (rat vs mouse hippocampus) or inefficient knockdown of SynDIG1 expression in hippocampal slice culture. It is also possible that the loss of SynDIG1 throughout development (as opposed to acute knockdown of SynDIG1 in slice culture) triggers a compensatory response to increase synapse number due to the overall weakening of SynDIG1-deficient synapses, as discussed above.

In summary, mice with reduced SynDIG1 exhibit deficits in the structure and function of excitatory synapses. Mature synapses are still present, albeit at a significantly reduced number, suggesting that synapses can reach maturity without SynDIG1. However, the overall reduction in the number of mature synapses by multiple criteria suggests that SynDIG1 is important for normal development. The increase in overall synapse number may be due to compensation for this loss of mature synapses or because SynDIG1-deficient synapses fail to be eliminated. Indeed, the increased response to glutamate uncaging demonstrates fundamentally altered excitatory synapse function in SynDIG1 mutant mice. Nevertheless, no candidate glutamate receptor subunits or MAGUKs appear to be altered in the composition of mutant postsynaptic densities, and AMPA/NMDA ratios are unaltered. Therefore, SynDIG1 appears to play an important role in the ability of excitatory synapses to reach or sustain maturity *in vivo* without gross changes to normal synapse composition.

## Synthesis

Reviewing Editor: Pablo Castillo, Albert Einstein College of Medicine

Decisions are customarily a result of the Reviewing Editor and the peer reviewers coming together and discussing their recommendations until a consensus is reached. When revisions are invited, a fact-based synthesis statement explaining their decision and outlining what is needed to prepare a revision will be listed below.

Your manuscript has been evaluated by two reviewers. The following is a synthesis of the online discussion on your study. The reviewers agree that your findings support a role for SynDIG1 in excitatory synapse maturation, although the study does not provide significant mechanistic insights and is therefore perceived as rather descriptive. They understand the conceptual advance is incremental, but still worth reporting as long as you properly address their concerns. They both agree that Fig. 1 does not add much to this paper, and that the quality of the results reported in Fig. 5 must be improved. As you will see in their comments to the authors, they also request clarification on a number of specific issues, including proper quantification of SynDIG1 in SynDIG1 beta-gal mutant mice. Regarding the statistical analysis, you should perform some normality test in order to use parametric tests. It is expected that the revision of your manuscript should take less than three months.
